# Femtosecond pulsed laser deposition of silicon thin films

**DOI:** 10.1186/1556-276X-8-272

**Published:** 2013-06-07

**Authors:** Matthew Murray, Gin Jose, Billy Richards, Animesh Jha

**Affiliations:** 1 Institute for Materials Research, Houldsworth Building, University of Leeds, Clarendon Road, Leeds, LS2 9JT, UK

**Keywords:** Femtosecond pulsed laser deposition, Silicon thin films, Chemical vapour deposition

## Abstract

Optimisation of femtosecond pulsed laser deposition parameters for the fabrication of silicon thin films is discussed. Substrate temperature, gas pressure and gas type are used to better understand the deposition process and optimise it for the fabrication of high-quality thin films designed for optical and optoelectronic applications.

## Background

Femtosecond pulsed laser deposition (fs-PLD) technique [1] uses a train of focused femtosecond laser pulses to generate plasma ablation from a target material; this plasma is deposited onto the surface of a substrate material, and the growth of a thin film occurs over time. The plasma itself consists of a mixture of ions and nanoparticles; at very high laser fluences, microparticles have also been observed [2]. This results in a thin film consisting of a solid state mixture of nanoparticles and occasionally microparticles. This makes fs-PLD an exciting nanofabrication technique with a considerable degree of variability in the fabrication process, still in the youth of its development.

The interaction of a femtosecond laser pulse with a target material has been experimented with and discussed by many [1-5], providing an in-depth view of the process and a wonderful demonstration of some of the fundamental physics involved. Firstly, we take silicon as an example of a target material; should a regular continuous wave laser be focused onto its surface, with an arbitrary energy just above its bandgap, one would observe the excitation of electrons to the conduction band through an indirect process involving phonons. This is because silicon has an indirect bandgap; one must use a wavelength of approximately 360 nm (3.43 eV) to trigger direct electronic excitation of silicon. A common laser wavelength for fs-PLD is 800 nm, only moderately above the bandgap of bulk crystalline silicon and so one would not expect significant ablation; however, femtosecond pulsed lasers are incredibly intense, and therefore, absorption occurs both by linear and nonlinear mechanisms [5]. Upon the excitation of an electron from the target material to the conduction band, in very high laser light intensities ( >10^13^ W/cm^2^) [6], a second photon can be absorbed by this electron and trigger avalanche ionisation, a nonlinear absorption process. Nonlinear absorption results in absorption increasing exponentially with respect to intensity. This ultimately gives rise to the majority of absorption of fs-laser pulses occurring in much shallower depths of the target than one would otherwise expect [7]. The absorption of the initial part of the femtosecond laser pulse thus gives rise to the formation of an electron-hole plasma in a relatively cold lattice of ions, and then, the rest of the pulse is absorbed through nonlinear mechanisms in the top surface of the material.

The laser pulse (100 fs) is much shorter than any electron-phonon relaxation mechanisms and hence does not interact with any heated material [5]. For silicon, relaxation processes are dependent on the electron-phonon coupling constant (1 ps for silicon); therefore, a dramatic increase in temperature occurs after this point. The temperatures experienced by the irradiated target area during fs-PLD are typically above that of the boiling point, depending on the fluence of the laser [2]. For a silicon target, there are certain thresholds associated with ablation from its surface. With an 800-nm wavelength and 80-fs pulse duration, Bulgakov et al. [8] demonstrated the emission of clusters (ionic and neutral) as well as singular ions and atoms (collectively, these shall henceforth be referred to as clusters) being emitted from a silicon target surface occurring at fluences as low as 100 mJ cm ^−2^ and increasing in yield with fluence. As the fluence is increased still further, a second threshold is reached, where nanoparticles of the target material begin to be ablated in tandem with the initially emitted clusters. The exact mechanism for the ejection of nanoparticles and microparticles from the target material is still under debate by many [1-5,8].

When compared to standard fabrication techniques such as chemical vapour deposition (CVD), a common technique for the fabrication of thin film and multilayered devices, fs-PLD offers a huge amount of versatility. CVD is often limited by the reactants used which are also commonly found to be either toxic, highly flammable or both. fs-PLD is not limited by the type of material either as ablation occurs via nonlinear absorption of the laser pulses; therefore, target materials as varied as glass, polymer, semiconductor, metal, etc. can be adopted to grow multilayered nanoparticulate thin films. It is important to note that target materials can also comprise any number of different elements, and all will be ablated without overly complex control of the experimental parameters, beyond that described earlier.

As described earlier, fs-PLD has the potential to be an extremely effective nanofabrication technique and therefore is worthy of exploration for its ability to fabricate solid state nanoparticulate thin films. Here, some of the defining parameters of fs-PLD are explored so as to fabricate high-quality devices with a smooth continuous deposited layer which is currently lacking in the literature. The optimised fabrication processes presented here has been utilised for Tm ^3+^-doped Si with successful room temperature emission from the ^3^F_4_ →^3^H_6_[9]. The use of silicon as an optical host material is also very attractive due to its large optical window in the infrared (IR) between 2 and 7 *μ*m. This IR region holds particular interest for identifying the molecular fingerprints of certain molecules and can also be utilised for optical communications.

## Methods

The fs-PLD fabrication process was done using a Coherent Ti-Sapphire LIBRA Laser (amplified 800 nm, 100 fs, 1 mJ, 1 kHz repetition rate; Santa Clara, CA, USA) focused at an angle of 60° from the normal surface of a selected target. A 50-mm diameter single crystalline silicon wafer was used as the target material and rotated/translated to avoid the formation of deep pits during ablation. The first set of experiments to analyse particle size was conducted by ablating the Si target for a short 2-min deposition time and collecting the ablated material onto a transmission electron microscopy (TEM) grid, which could then be analysed using a Phillips FEI Technai TF20 field emission gun TEM (Hillsboro, OR, USA) operating at a gun voltage of 200 kV. These samples were analysed by taking a series of images of different areas of the grids and measuring each particle diameter. The results are presented here as histograms for depositions made at 20, 40 and 60 mTorr as well as some accompanying TEM micrographs.

Following this, thin films were grown on fused silica substrates over a time period of 2 h in Ar or 4% H in Ar background gas in the range of 20 to 70 mTorr. The substrate was positioned 70 mm away from the target material and was rotated at a constant 20 rpm during the fabrication. These thin films were characterised by scanning electron microscopy (SEM) and Raman microscopy. These were done to carry out optical, electronic and structural analysis of the films to better define parameters for growing a film for optical applications and high-quality device fabrication.

## Results and discussion

### Sub-monolayer deposition

**Figure 1:**
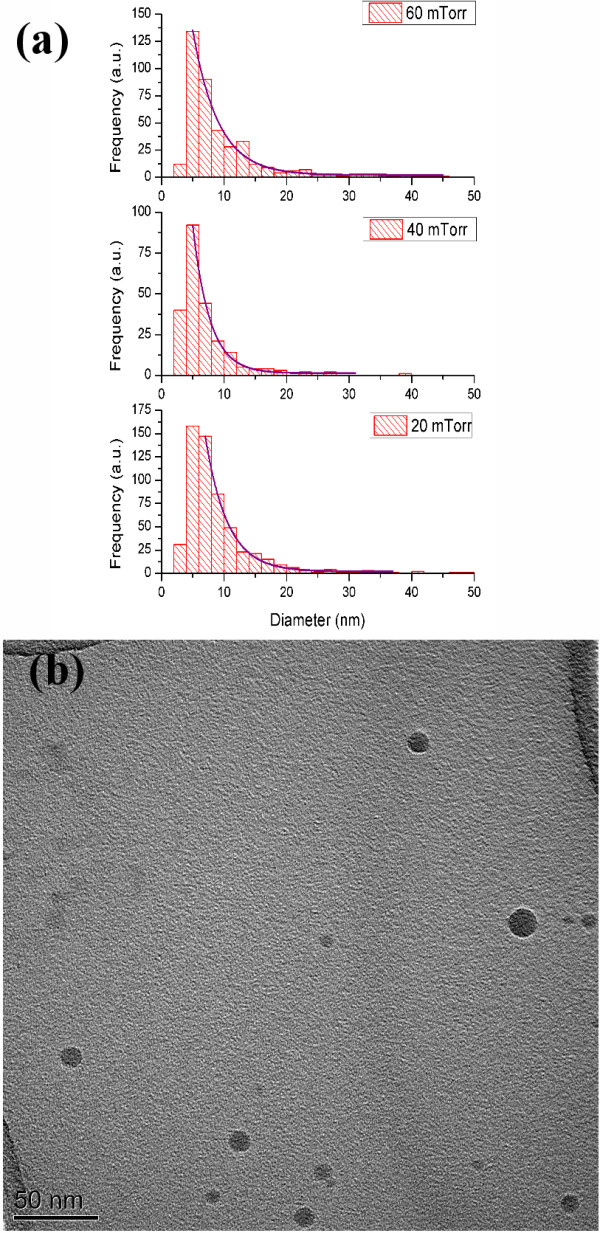
**TEM particle size analysis.** (**a**) Particle diameter histograms for samples deposited at 20, 40 and 60 mTorr with exponential decay fits (**b**) TEM micrograph of particles deposted at 20 mTorr in 4% H in Ar.

Figure 1a shows the exponential decay fit to the histogram of each background pressure, in good statistical agreement to the data itself. The fit has been limited to 6 nm diameters and above for particles deposited at 20 mTorr and 4 nm and above for 40 and 60 mTorr. This is because below this diameter, the resolution and contrast ratio of the particles with respect to the copper grid are too low for an accurate assessment of particle size. These results are in good agreement with observations by Amoruso et al. [10] in vacuum, where a similar exponential character was identified for the relative yield of particle sizes. Importantly, this is an indicator of the large abundance of silicon nanoparticles below the exciton Bohr radius and can therefore be considered as quantum dots.

For the growth of continuous thin films, fabricated by fs-PLD, it is necessary to include some sort of background gas to widen the plasma plume and therefore evenly deposit over a substrate surface. Without a background gas, the plasma plume will be very narrow and thus form a very uneven film surface from one side to the other, where the majority of deposits will be made in the very centre [11]. A background gas decreases the kinetic energy of the ablated particles and causes gradual changes in their trajectory with subsequent collisions. One foreseeable consequence of the addition of a background gas is the agglomeration of ablated particles. This however is not observed for the present study because the effective temperature of the ablated material is too high, and the strong positive ionisation of many of the particles would inhibit the formation of aggregates. This is why only isolated particles are observed in the TEM micrograph of Figure 1b and others.

Microparticles were not observed during the TEM analysis; however, the large abundance of nanoparticles indicate that the laser parameters are well within the second threshold of ablation, i.e. only clusters and nanoparticles will be ablated. This is an ideal regime to work in for high-quality optical materials because of the expected decrease in surface roughness and a better continuity throughout the film. One could however expect some microparticles to become ablated over lengthy deposition times (e.g. 2 h or more) due to extensive surface modification of the target material [6].

### Thin film deposition

Silicon thin films were grown to investigate microstructural quality and to determine whether they are suitable for optical applications and subsequent device fabrication. These were fabricated under the same laser parameters as used in the sub-monolayer analysis at 20 mTorr background gas pressure, where a greater abundance of silicon quantum dots are ablated. In order to assess the experimental parameters best suited for the fabrication of thin films, an array of samples was fabricated under varying conditions, and some of the conclusions identified are presented here. The primary variables analysed in this study are the temperature of the substrate during the deposition, the laser fluence, the background pressure and the background gas type used.

**Figure 2:**
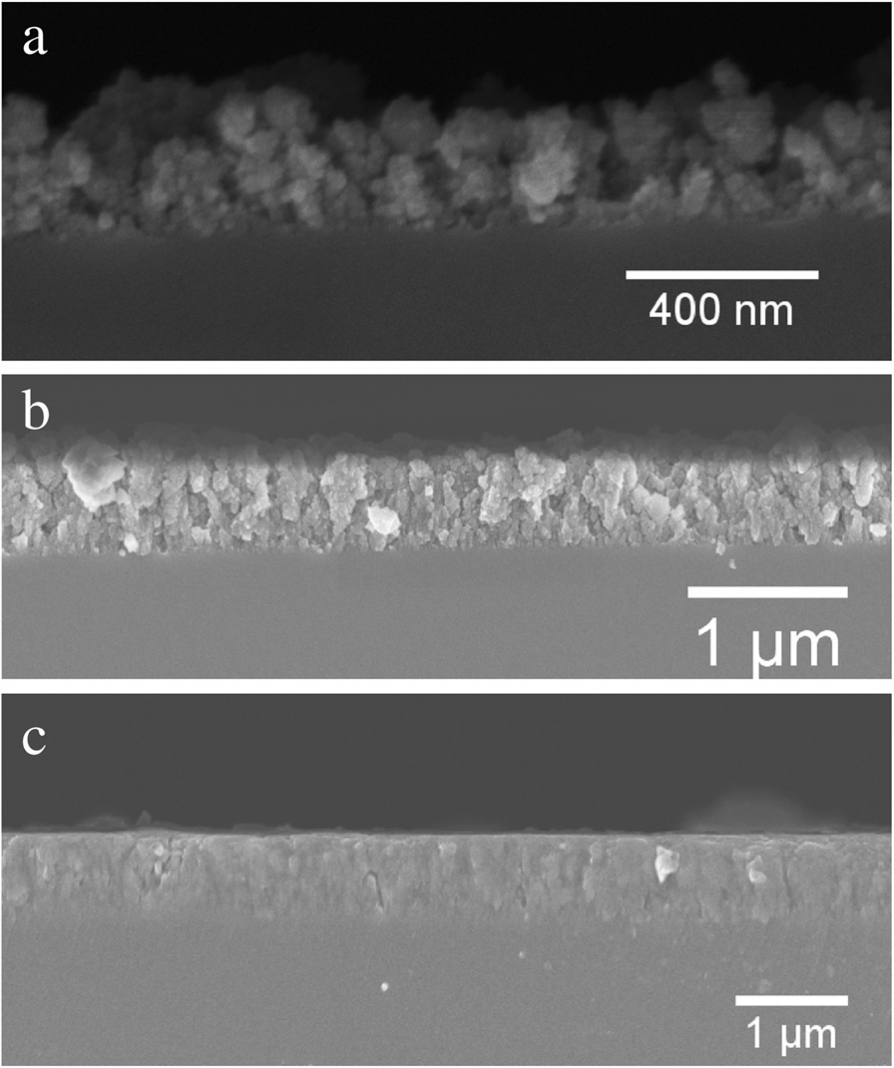
**SEM cross sections of Si thin films fabricated under different deposition parameters.** SEM cross sections of Si thin films deposited by (**a**) room temperature in an Ar atmosphere, (**b**) room temperature in 4% H in Ar and (**c**) 200°C in 4% H in Ar.

Presented in Figure 2 are SEM cross sections of selected thin films deposited under different parameters: (a) deposited at room temperature in Ar, (b) deposited at room temperature in 4% H in Ar and (c) deposited at 200°C in 4% H in Ar. The cross sections of these samples were prepared by scouring the back of the silica substrates and then snapping along that line, taking adequate measures to ensure the film is not damaged in the process. This produces a clean break along the length of the sample where a cross section of the thin film can be seen clearly. From the presented images of Figure 2, it is clear that there is a considerable degree of variability in the density and morphology of the deposited films with regard to the deposition parameters. A key observation for the ablation of Si in either Ar or 4% H in Ar is that the addition of hydrogen to the argon gas considerably reduces the surface roughness and the appearance of cauliflower-like surface features (see Figure 2a). The observed increase in the smoothness of the film due to the inclusion of hydrogen in the background gas is believed to stem from an enhancement of the transferral of kinetic energy from the ablated material through collisions with both hydrogen and argon. Greater momentum transferral can therefore occur to hydrogen and therefore better disperses the plasma plume. A smooth surface and continuous film depth profile are important for both the fabrication of multilayered functional devices and for electrically conductive materials. The inclusion of hydrogen in the background gas, as demonstrated here, can therefore be viewed as an important experimental parameter for the development of such materials and devices.

**Figure 3:**
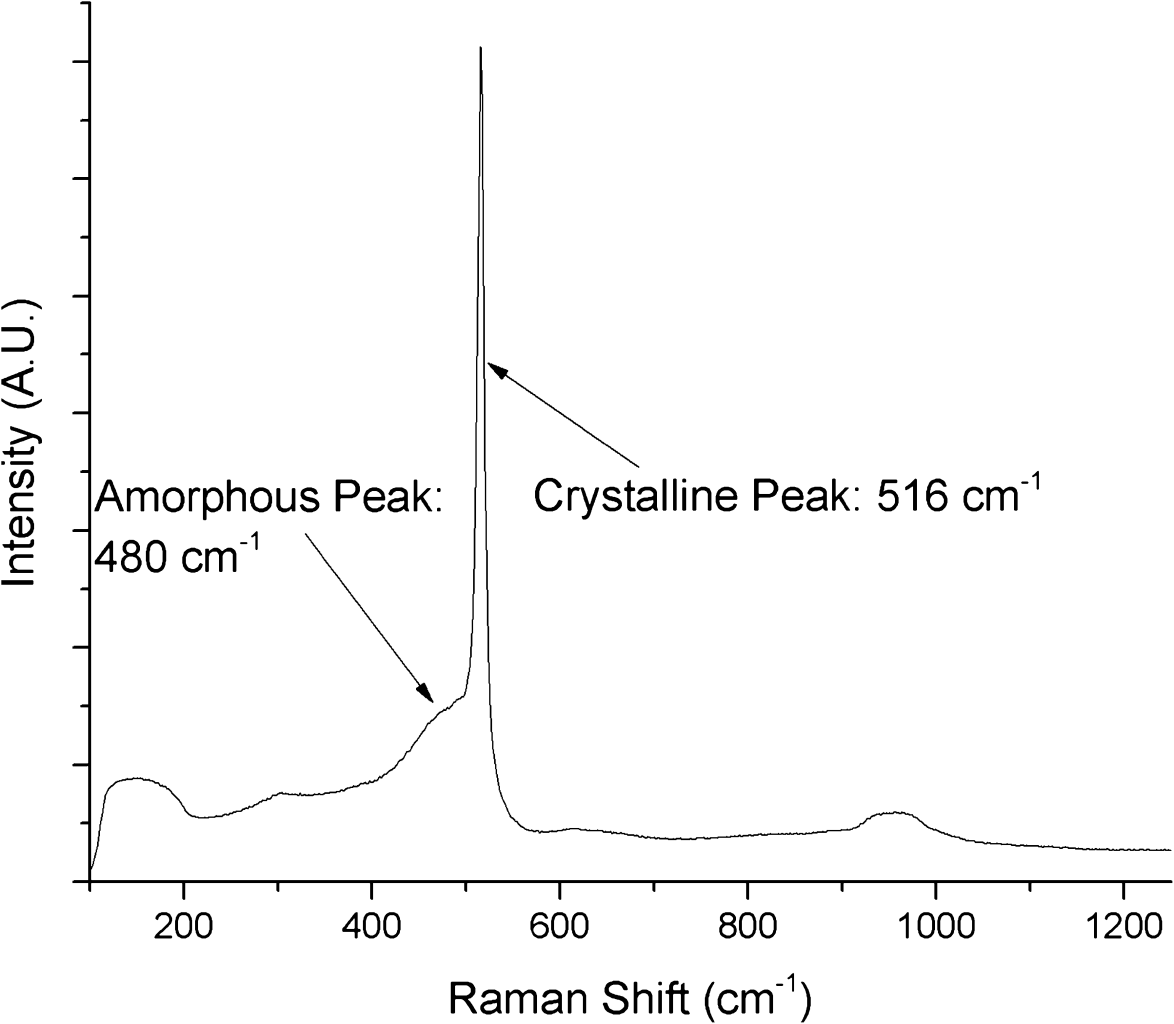
**Micro-Raman spectroscopy of sample deposited at 200°C.** Crystalline fraction found at approximately 520 cm ^−1^ and the amorphous fraction at 480 cm ^−1^, demonstrating a mixture of the two phases within the films.

The heating of the substrate during the deposition of the sample presented in Figure 2c provides further information to the fabrication of thin films via fs-PLD. As can be seen, a noticeable reduction in pores throughout the film is observed, relative to Figure 2b, as well as maintaining the smooth surface. As discussed earlier, fs-PLD deposits a range of nanoparticulate sizes; for silicon, these particles can be either in a crystalline phase or an amorphous phase. Raman spectroscopy is commonly employed for the analysis of silicon nanoparticles; it is a powerful technique which can define the average particle size as well as give an indicator for the amorphous to crystalline ratio of the particles. In order to accurately define the average particle size, one must also take note of the stresses on the particles themselves; however, TEM analysis has already given the particle size distribution, and therefore, this will not be discussed here. Micro-Raman spectroscopy was carried out using a Renishaw InVia micro-Raman microscope (Wotton-under-Edge, UK) on several films and identified a mixture of amorphous and crystalline phases in the material. From Figure 3, one can see the sharp Lorentzian peak at 520 cm ^−1^ to signify the existence of crystalline silicon and the broad Gaussian peak at 480 cm ^−1^ which represents the amorphous fraction of the film.

**Figure 4:**
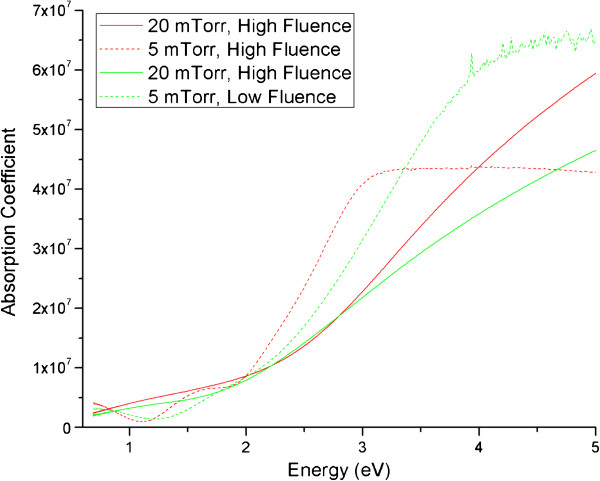
**Relationship between absorption coefficient and laser fluence and background gas pressure used during deposition.** Decreasing trend in absorption coefficient with respect to increasing gas pressure and decreasing laser fluence.

Optical transmission spectroscopy was also carried out to observe variations with regard to the absorption of films fabricated under different conditions. By varying the fluence of the laser and/or the background gas pressure in 4% H in Ar, a qualitative relationship was identified with regard to variations in the absorption coefficient of the materials. This is presented in Figure 4, where samples deposited at a lower fluence demonstrate an increased absorption coefficient and those deposited at 5 mTorr as opposed to 20 mTorr also demonstrate a higher absorption coefficient. These results are fit with the current theory because a higher fluence would increase the yield of nanoparticles being ablated and hence increase the overall absorption, particularly at lower wavelengths (higher energy). At lower pressures, a greater abundance of smaller nanoparticles has been observed in the sub-monolayer analysis in Figure 1, again increasing the absorption at higher energies; this is likely due to a decrease in redeposition of ablated material, and therefore, more nanoparticles are deposited on the substrate in lower pressures over time.

## Conclusion

To conclude, femtosecond pulsed laser deposition has been used to fabricate solid state nanoparticulate silicon thin films on a fused silica substrate. Fabrication parameters have been studied in order to form high-quality thin films with a continuous film profile and a smooth surface, ideal for optical and optoelectronic applications. The inclusion of hydrogen in a background gas of argon and the heating of the substrate during deposition have both been shown to dramatically improve the as-deposited film quality. To further this work, it would be appropriate to carry out a quantitative assessment of how properties such as the emission characteristics from a doped lanthanide or the electrical conductivity would vary depending on the fabrication processes described above. The conclusions drawn here are also not limited to the fabrication of silicon thin films but can be utilised for better refining the deposition process of different materials.

## Competing interests

The authors declare that they have no competing interests.

## Authors’ contributions

MM fabricated each sample, and all authors (MM, GJ, BR and AJ) assisted in analysing the data. MM prepared the figures and manuscript. All authors are aware of the article and consent to its publication. All authors read and approved the final manuscript.
